# Effects of inulin diet supplementation on production performance, gut traits, and incidence of ascites in Haidong chicks under hypoxic conditions

**DOI:** 10.5713/ajas.20.0508

**Published:** 2020-10-14

**Authors:** Baoan Ding, Lingyun Chen, Hao Lin, Xiezhong Wang, Licheng Zhang, Xiaoming Ni, Andrea Pirone, Stephen R. Madigosky, Baldassare Fronte

**Affiliations:** 1 State Key Laboratory of Plateau Ecology and Agriculture, College of Agriculture and Animal Husbandry, Qinghai University, Xining 810016, China; 2 Qinghai Animal Disease Control Center, Xining 810001, China; 3 Dipartimento di Scienze Veterinarie, Università di Pisa, Viale delle Piagge 2, 56124, Pisa, Italy; 4 Department of Environmental Science and Biology, One University Place, Widener University, Chester, PA 19013, USA

**Keywords:** Haidong Chicks Performance, Ascites, Gut Traits, Inulin, Nutrition

## Abstract

**Objective:**

Effects of inulin supplementation in diet of Haidong chicks under hypoxic conditions on production performance, intestinal morphologic change, microflora contents and the incidence of ascites were studied.

**Methods:**

Commercial male chicks (360) were randomly divided into 6 groups and were fed diets supplemented with 0, 0.05, 0.075, 0.1, 0.125, and 0.15 g/kg of inulin, respectively.

**Results:**

The body weight gain and feed intake were improved in chicks fed the diets supplemented with 0.1 and 0.125 g/kg of inulin, from d 1 to d 42 (p<0.05); moreover, blood parameters were positively affected when inulin was included in the diets and the thickness of the intestinal wall and muscle tissue in duodenum, jejunum, and ileum tended to increase (p<0.05), and the villi height and crypt depth in duodenum, jejunum, and ileum (p<0.05). Regarding the number of goblet cells in duodenum, jejunum and ileum tended to increase when chicks were fed the diets supplemented with 0.075, 0.1, 0.125, and 0.15 g/kg (p<0.05) of inulin. When chicks were fed diets supplemented with 0.75 or 0.1 g/kg of inulin, a significant reduction of *Escherichia coli* counts in the cecum was observed; for a contrary, a significant increment of *Bifidobacterium* and *Lactobacillus* was observed in cecum and ileum. Finally, supplementing the feed with inulin determined an overall reduction of ascites incidences in comparison to the control group.

**Conclusion:**

Thus, the results observed in the present study clearly suggest that the diet supplementation with a quantity of inulin ranging between 0.1 and 0.125 g/kg, can improve growth performances, intestinal morphology, internal microbial balance and ascites incidence, in broiler chicks raised at high altitude area. Even though these findings may be of interest for the poultry industry, they may particularly be relevant in those areas characterized by high altitude such as Northwest China regions.

## INTRODUCTION

Prebiotics are defined as non-digestible food ingredients that can benefit the host by selectively stimulating the growth and activity of supportive microorganisms while limiting the total number of bacteria in the large intestine [1,2]. Oligosaccharides that are not hydrolyzed by digestive enzymes in the upper digestive tract of a host, enter in the hindgut where they are fermented by the intestinal microbiota. Inulin-type prebiotics are members of a larger group called “fructans” [2,3]. These consist in a category of compounds that include all naturally occurring plant oligo and polysaccharides characterized by one or more fructosyl-fructose linkages and glycosidic bonds; thus, they primarily are polymers of fructose units. Inulin is a group of naturally occurring polysaccharides produced by many types of plants [3] even though it is industrially are mainly extracted from chicory and used as a prebiotic to improve gut health and performances in animals [4–7].

The administration of dietary prebiotic and probiotic to animals may produce metabolic changes at several levels. For example, at blood level, many recent papers refer that probiotics reduce serum cholesterol (CH) levels and increase packed cell volume (PCV), hemoglobin (Hb), and red blood cell (RBC) values [8]. In relation to the use of a prebiotic product, Piray and Kermanshahi [9] refer that no significant differences in total CH, highdensity lipoprotein (HDL), low density lipoprotein (LDL), and triglycerides (TG) where observed when a prebiotic product was included in the diet. Differently, the immunoglobulin G (IgG), IgA, IgM serum levels were significantly decreased in broilers fed with prebiotic [9,10].

At gut level, it is assumed that an increased villus height is paralleled to an increased digestive and absorptive function of the intestine, due to increased absorptive surface area, expression of brush border enzymes, and nutrient transport systems [11,12]. The mode of action of prebiotics on morphological parameters, to a great extent is indirectly influenced by alterations in microbial fermentation patterns in the gastrointestinal tract [3]. Consequently, feeding inulin to broilers may increase the absorption of nutrients by improving the intestinal mucosal structure such as increasing the villus height and deeper crypts [13,14]. Moreover, the inclusion of inulin in diet for piglets has shown a significant increase of neutral goblet cells in ileal villi, whereas it determined a reduction of acidic goblet cells density in jejunum villi [15]. Recently, several authors suggested that the use of prebiotics is a promising approach to enhance the role of beneficial endogenous organisms located in the gut. Tako and Glahn [16] reported that the proportions of bifidobacterium and lactobacilli in the intestinal contents of chickens fed a diet of inulin were substantially higher. Most studies that evaluate the presence of bacteria in the small intestine of monogastric animals have shown that bacteria are capable of utilizing indigestible carbohydrate sources for energy. An understanding of the phenomena may help to explain how animals, especially chickens, react to hypoxic conditions as displayed by the condition known as ascites. To this regard, Solis de los Santos et al [17] reported that the addition of a prebiotic to broiler diet improves gut development in neonates, with that inducing an increased enteric efficiency that may lead to a reduction of the gastro intestinal tract (GIT) oxygen demands and to an alleviation of factors leading to ascites.

Ascites is a metabolic disease characterized by an accumulation of fluid in the abdominal cavity, hypertrophy of the right ventricle, and a flaccid heart [18]. A high incidence of ascites occurs when broilers are reared at high altitudes owing to the reduction of the partial pressure of oxygen [18]. This often results in a substantial economic loss and one that needs to be addressed using a variety of remedies such as the use of prebiotics. Solis de los Santos et al [17] showed that broilers fed prebiotic supplements under hypoxic conditions, had a 23% lower incidence of ascites compared with control birds maintained in the same environment. The GIT is a metabolically active organ that has considerable nutrient and oxygen requirements [18]; it is intimately linked to the cardiopulmonary system and both dependent upon each other; however, this relationship can be negatively influenced by several factors such as inflammations, pathogens, an elevated metabolic rate or a stressful environment, thus resulting in ascites onset [18].

Although, there are considerable researches addressing the use of prebiotic supplements in broilers nutrition [19,20], still no available information regarding effects of inulin on the intestinal morphology and microflora in broilers under in high altitude condition. For this reason, the purpose of the present study was to investigate possible effects of inulin supplementation in diet of Haidong chicks under hypoxic conditions on production performance, intestinal morphologic change, microflora contents and the incidence of ascites.

## MATERIALS AND METHODS

### Bird management and treatments

All procedures were performed in accordance with institutional guidelines set for the care and use of laboratory animals prescribed by Qinghai University. All experiments were implemented in Huzhu county of the Qing-zang high altitude region (2,880 m) of western China (36°42′37″N, 102°10′57″E). A total of 360, one-day old Haidong chicks (Qinghai Huzhu xueling breeding farm, Huzhu, China) were randomly distributed into six treatments (groups) and six replicates, 10 birds per replicate. To the diet fed to the control group (C) no inulin was added; for a contrary, to the diet fed to the remaining groups (I1, I2, I3, I4, I5) 0.05, 0.075, 0.1, 0.125, and 0.15 g/kg inulin (CAS NO. 39289-43-5, Yang Ling Ciyuan Biotechnology Co. LTD, Xi’an, China) was added, respectively. All groups received a diet formulated to meet the standard nutrient requirement suggested for the AVIAN500 commercial broiler chicks (Beijing Poultry Breeding Company Ltd, Beijing, China, 2005) and the diet compositions are shown in Table 1. The ingredients and chemical compositions of the diets were analyzed using an AOAC (2000) protocol. Chicks were housed in stainless-steel wire floor cages and battery brooder pens. The room temperature was maintained between 30°C and 32°C from day 1 to day 14, between 24°C and 26°C from day 15 to day 21 and between 22°C and 24°C from day 22 to 42. A 5 lux incandescent bulbs supplied light (23-h light and 1-h dark schedule) to the experimental room over the duration of the study. Food and water were provided *ad libitum*. Body weight and feed intake were recorded on a weekly basis.

### Blood analysis

After 6 h of fasting, on day 21st and 42nd one bird from each replicate was randomly chosen and blood samples taken from the brachial vein; blood samples were collected into both heparinized test tubes and plain non-heparinized tubes; the heparinized blood samples were centrifuged (2,000 rpm for 10 min) for serum separation while the blood in plain non-heparinized tubes was directly stored at −20°C until assayed. The following blood parameters were measured: CH, TG, HDL, LDL, PCV, RBC count, white blood cell (WBC) count and Hb. The index of PCV, RBC and WBC were determined by automated hematology analyzer (Sysmex KX-21, Kobe, Japan). Hb concentration was calculated by Cyanmet-hemoglobin method. The concentration of CH, TG, HDL, and LDL were estimated according to the procedures recommended by the manufacturer of the diagnostic kits (BioSystems, S.A. Barcelona, Spain) and spectrophotometer apparatus.

### Histological analysis of the intestinal morphology

To determine the effect of inulin on intestinal morphology, six birds from each group were randomly selected and sacrificed by cervical dislocation on day 42. Segments of approximately 5 cm of duodenum (midpoint of the pancreatic loop), jejunum (midpoint of jejunum) and ileum (after Meckel’s diverticulum) were obtained before the removal of the entire intestinal tract. Samples were fixed in 4% buffered formalin solution for 24 h and processed for routine paraffin embedding. Serial 4 μm transverse sections were cut and stained with haematoxylin and eosin for morphological evaluation. Determination of goblet cells containing acidic and neutral mucin was performed by staining sections with alcian blue (AB) pH 2.5 + periodic acid-Schiff reagent (PAS). Sections were examined using a light microscope (Leitz, Diaplan, Leitz, Stuttgart, Germany) connected to a PC via a Nikon digital system (Digital Sight DS-U1, Nikon, Tokyo, Japan). Images were acquired using the NIS-Elements F version 2.10 software per transverse section of small intestine. Measurements were made using Image J 1.37V software. Ten well-oriented and intact crypt-villus units of each slide were measured in triplicate. The villi height was defined as the distance from villus tip to crypt junction. The crypt depth was defined as the depth of the invagination between adjacent villi. The muscle thickness was measured from the junction between the sub-mucosal and muscular layers to that between the muscular layer and the tunica serosa. The thickness of intestinal wall was defined as the distance from intestinal external subserosa to the muscular junction layer and submucosa. The number of AB/PAS-positive cells along the villi determined the density of cells per mm^2^.

### Intestinal microflora

To determine the effect of inulin on the intestinal microbiome, at 42 days 6 samples of cecum and ileum content from each group were collected for analysis of the microbiota. One gram of cecum and ileum contents was blended in 9 mL of sterilized physiological saline solution and homogenized using a bag mixer. Subsequently, the suspensions were serially diluted from 10^−1^ to 10^−6^ using sterilized physiological saline solution. Duplicate plates per sample were inoculated with 100 μL of suspension and anaerobically incubated at 37°C.

### Cultivation and enumeration of bacteria

*Lactobacillus* was anaerobically assayed using lactobacilli MRS agar (Fisher Scientific, Ottawa, ON, Canada) and incubated at 37°C for 48 h. A count of bifidobacteria was performed using Wilkins-Chalgren agar (Oxoid, Nepean, ON, Canada) supplemented with glacial acetic acid (1 mL/L) and mupirocin (100 mg/L) extracted from antimicrobial discs (Oxoid, London, UK) and incubated at 37°C for 20 h. *Escherichia coli* were cultured using MacConkey’s agar medium (Oxoid, UK). Numbers of colony-forming units from duplicate plates per bird sample were averaged and the results were expressed as log colony-forming units per gram of intestinal content.

### The incidence of ascites

To determine the effect of inulin on the incidence of ascites, a protocol described by Pavlidis et al [21] was used; according to the method, chickens with a right ventricle/total ventricle ratio over 0.27 were considered ascitic. To this purpose, the evaluation was performed on 12 chicks from each group. As a result of the ascites condition, mortality was recorded on d 42 of the experiment.

### Statistical analysis

Mean values generated from all individual data were statistically analyzed by a one-factor variance analysis using the general linear model procedure of the SAS Institute (Cary, NC, USA). If the main effect between any group was found to be significant, they were then compared using the Tukey’s multiple range test at p<0.05. The statistical evaluation of the incidence of ascites between treatment and control groups was performed using the Person’s Chi-square test, once main effects were significant with p<0.05.

## RESULTS

In relation to the growth performances (Table 2), on day 42 a higher body weight and body weight gain were observed for the groups fed 1.0 (I3) and 1.25 g/kg (I4) inulin compared to all the other groups (p<0.05); however, even the group fed 0.75 g/kg inulin (I2) showed higher values (p<0.05) than Control and I1 group. In relation to feed intake, the highest value was observed for chickens fed 1.0 g/kg inulin (I3), significantly different in comparison to all other considered groups (p<0.05); at the same time, the animals fed 0.75 g/kg (I2) and 1.25 g/kg (I4) showed higher (p<0.05) feed intake than the those fed 0.05 (I1) and no inulin (Control group). Despite that, no significant difference (p>0.05) between groups was observed for feed conversion ratio.

The effect of inulin on PCV, RBC, WBC, and Hb, are described in Table 3. On day 21 PCV values in group I4 with 0.125 g/kg inulin were higher (p<0.05) than in 0.15 g/kg (I5) and control group (C); at the same time, the groups fed doses between 0.05 g/kg and 0.1 g/kg (I1, I2, and I3) showed higher (p<0.05) values than control group (C), but not significantly different than the values observed for the group fed higher doses of inulin (I4 and I5). On day 42, the PCV values observed for the groups fed 0.05 and 0.125 g/kg of inulin (I1 and I4, respectively) were higher (p<0.05) than those observed for the groups fed 0.15 g/kg (I5) or no inulin (C) while no significant differences were observed with the remaining considered groups.

On day 21, the groups fed 0.15 and 0.1 g/kg (I5 and I3, respectively) showed highest (p<0.05) number of RBC; moreover, RBC value was higher (p<0.05) for groups fed 0.05 and 0.125 g/kg of inulin (I1 and I4, respectively) in comparison to groups fed 0.075 g/kg (I2), and this latter higher (p<0.05) than control group (C). At 42 days the number of RBC observed for the broilers fed 0.05 and 0.15 g/kg inulin (I1 and I5, respectively) was significantly higher (p<0.05) than that observed for the broilers fed 0.1 g/kg (I3); similarly, the RBC values observed for this latter group and for the groups fed 0.075 and 0.125 g/kg (I2 and I4, respectively) were also significantly higher than that observed for control group (C).

On day 21, WBC values were higher (p<0.05) for the group fed 0.125 g/kg inulin (I4) in comparison to the groups fed 0.05, 0.075, and 0.15 g/kg (I1, I2, and I5, respectively), as well as to the group fed no inulin (C); statistically significant differences were also observed for the group fed 0.1 g/kg of inulin (I3) in comparison to the group fed 0.15 g/kg (I5) and the control group (C). Despite these early differences observed on day 21, for WBC number no difference was observed on day 42 (p>0.05).

In relation to Hb values on day 21, the broilers fed 0.125 g/kg (I4) of inulin showed a higher value (p<0.05) than all the other groups, except for group I5 fed 0.15 g/kg (p>0.05); however, this latter group showed a higher (p<0.05) Hb value than 0.05 g/kg (I1), 0.1 g/kg (I3) and control group (C); furthermore, statistically significant differences (p<0.05) were also observed between the animals fed 0.05 g/kg and 0.1 g/kg of inulin (I1 and I3, respectively) and the control group (C). On day 42, the groups fed 0.125 and 0.15 g/kg of inulin (I4 and I5, respectively) showed higher Hb values than the groups fed 0.05 and 0.1 g/kg of inulin (I1 and I3, respectively); finally, the Hb values of these latter groups, together to the group fed 0.075 g/kg of inulin (I2) were also higher (p<0.05) than the values observed for control group (C).

In relation to the thickness of the intestinal wall (Table 4), the highest value in the duodenum was observed in chickens fed 0.1 g/kg (I3) inulin in comparison to all the other considered groups (p<0.05); thus, birds fed 0.075 (I2), 0.125 (I4), and 0.15 (I5) g/kg inulin, showed a significantly thicker intestinal wall than those fed no (C) or 0.05 g/kg (I1) inulin (p<0.05). In the jejunum, the wall thickness was significantly higher (p<0.05) in birds fed 0.1 (I3), 0.125 (I4), and 0.15 g/kg (I5) dietary inulin, in comparison to those fed 0.05 (I1), 0.075 (I2) and no inulin (C). In the ileum, the thickness of the intestinal wall was higher (p<0.05) when 0.1 (I3) and 0.15 g/kg (I5) of inulin was added to the diet in respective to the birds fed 0.05 (I1), 0.075 g/kg (I2), and no inulin (C); for a contrary, no differences (p>0.05) were observed between birds fed 0.1 (I3) and 0.15 (I5) in comparison to those fed 0.125 g/kg (I4) inulin even though these latter birds showed a higher (p<0.05) wall thickness than the chickens fed no inulin (C) or 0.05 g/kg (I1) only. In addition, the ileum intestinal wall thickness was higher (p<0.05) in the birds fed 0.075 g/kg (I2) dietary inulin in comparison to those fed 0.05 g/kg (I1) inulin.

The intestinal muscle thickness (Table 4) in the duodenum was higher (p<0.05) in groups fed 0.1 (I3), 0.125 (I4), and 0.15 g/kg (I5) inulin in comparison to the remaining groups; among these latter, differences (p<0.05) were unexpectedly observed between birds fed no (C) or 0.075 g/kg (I2) inulin in comparison to those birds fed 0.05 g/kg (I1). At jejunum level, the greatest and significantly different intestinal muscle thickness was observed in birds fed 0.1 g/kg (I3) dietary inulin (p<0.05) in comparison to the other groups; thus, the birds fed 0.125 (I4) and 0.15 g/kg (I5) of inulin, showed a higher (p<0.05) intestinal muscle thickness than those birds fed no (C) inulin or just 0.05 (I1) and 0.075 g/kg (I2). At ileum level, the highest intestinal muscle thickness was observed in chickens fed 0.075 g/kg (I2) inulin, significantly different (p<0.05) solely in comparison to the chickens fed no inulin (C).

Regarding the intestinal villus height (Table 5), birds fed the diets containing 0.1 g/kg (I3) inulin showed the highest villi in comparison to all the other groups; meantime, birds fed 0.075 (I2), 0.125 (I4), and 0.15 (I5) showed higher villi than the remaining birds (I1 and C). The jejunum villi of birds fed diets containing 0.1 (I3), 0.125 (I4), and 0.15 g/kg (I5) inulin were higher than those fed 0.05 (I1) and 0.075 g/kg (I2) dietary inulin (p<0.05); at the same time, these latter birds showed higher (p<0.05) villi than birds fed no inulin (C). At ileum level, villi of birds fed diets containing 0.1 (I3), 0.125 (I4), and 0.15 g/kg (I5) inulin showed higher villi than those fed 0.075 g/kg (I2), this latter being higher (p<0.05) than those of birds fed no (C) or 0.05 g/kg inulin.

Similar results were observed for crypt depth (Table 4) and at duodenum level birds fed 0.075 g/kg inulin or more (I2, I3, I4, and I5) showed deeper crypts than the birds fed lower doses of inulin (C and I1). At jejunum level, birds fed a higher dose than 0.1 g/kg of inulin (I3, I4, and I5), showed deeper crypts (p<0.05) solely in comparison to birds fed no inulin (C). Finally, similar results were observed at ileum level; in fact, at this level doses higher than 0.05 g/kg inulin (I2, I3, I4, and I5) determined deeper crypts (p<0.05) than the lower inulin doses (I1 and C). While the inulin in diet did not affect the Villus height/villus crypt values in the duodenum, jejunum, and ileum of chickens (p>0.05).

The number of goblet cells (Table 6) in duodenum were significantly increased (p<0.05) when broilers were fed diets containing 0.1 g/kg or more inulin (I3, I4, and I5) compared to those fed a diet containing 0.05 g/kg (I1); however, for this latter inulin inclusion level, a significantly higher (p<0.05) goblet cell density was observed in comparison to control group. In the jejunum, the higher (p<0.05) goblet cell densities were observed when inulin was included at a concentration of 0.1 g/kg or higher (I3, I4, and I5); however, when inulin was feed included at a concentration of 0.05 (I1) or 0.075 g/kg (I2), goblet cell densities were significantly higher (p<0.05) than that of the control group (no inulin inclusion). At ileum level, the highest goblet cell density was observed for inulin inclusion equal to 0.1 g/kg (I3) in comparison to the other lower inclusion rates (I2, I1, and C); moreover, differences were significant even between the group fed 0.075 (I2) and 0.05 (I1), and between this latter and control group (C).

Finally, regarding the Ascites Incidence, the highest values (p<0.05) were observed for the broilers fed no inulin (C), while the considered inulin inclusion rate did not affect the ascites incidence (p>0.05).

The dietary inulin inclusion at a concentration of 0.075 (I2), 0.1 (I3), and 0.15 g/kg (I5) significantly decreased (p< 0.05) the concentration of *Escherichia coli* in the cecum (Table 7); unexpectedly, the addition of 0.125 g/kg of inulin, did not show a significant effect (p<0.05) in comparison to the birds fed no inulin or 0.05 g/kg. For a contrary, at cecum level, the concentrations of *Bifidobacterium* were significantly increased by the administration of 0.075 (I2), 0.1 (I3), and 0.125 g/kg (I4) inulin (p<0.05), but only in comparison to the control group that was fed no inulin. Finally, independently of the diet inclusion rate, inulin significantly increased the *Lactobacillus* population (p<0.05). Conversely, the concentration of *Escherichia coli* at ileum level was not affected by the dietary administration of inulin (p>0.05), while the concentrations of *Bifidobacterium* were increased (p<0.05) when the inulin inclusion rate was 0.125 (I4) and 0.15 g/kg (I5), in comparison to the control group (C) only. As already observed for the cecum, inulin positively affected (p<0.05) the Lactobacillus concentration at ileum level, independently of its diet inclusion rate.

## DISCUSSION

The results of the present study clearly showed that including inulin in diets for Haidong chicks was beneficial for body weight gain; to this regard, the most effective levels of inulin inclusion were 0.075, 0.1, and 0.125 g/kg. Aluwong et al [22] refer about similar results on body weight when yeast and yeast derived products were included in diets for broiler chickens. Concerning the average feed intake, the birds fed inulin (0.075, 0.1, and 0.125 g/kg) consumed significantly more feed than both broilers fed no inulin (C) and those receiving 0.05 g/kg (I1). These findings are consistent with numbers of experimental studies, which refer that including yeast derived products or prebiotics may increase feed intake of broiler chickens [1,23]. However, no significant effect of inulin inclusion was observed for feed conversion rate.

In the present study, blood parameters (PCV, RBC, WBC, and Hb) were positively affected because of the addition of inulin in the diet of Haidong chicks. These results agree with those observed in a study carried out by Al-Kassie et al [8], where the diet inclusion of a prebiotic showed similar results. Hence, since when hypoxia occurs broilers encounter difficulties meeting tissues oxygen demand, it is reasonable to assume that prebiotics enhance growth performances by improving blood saturation, RBC production (partially immature) as well as hemoglobin content [24]. Despite that, Samolińska et al [25] observed that prebiotic diet supplementation did not cause significant increase in the erythrocyte count, hemoglobin concentration and hematocrit values of broilers; probably, the different results observed may be due to the characteristics (type and number of species) of prebiotics.

The histological evaluation of the intestinal morphology is considered a valuable tool in the assessment of modifications induced by several factors and, in particular, different dietary regimes [23,26]. Hence, this approach was used in the present study and it highlighted a positive effect of the inclusion of inulin in the chick diets. In fact, results showed that after 42 days of treatment, gut traits were significantly improved. Specifically, the thickness of intestinal wall and muscle layer in duodenum, jejunum and ileum were increased when chicks were fed 0.1 and 0.125 g/kg of inulin; these results are in agreement with those described by Solis de los Santos et al [17] about the thickness of the lamina propria of duodenum and ileum, observed when chicks were fed prebiotic. The thickness of both intestinal wall and muscle layer, are suggested as an effective indicator of gut health, since they host dendritic cells that have a wide array of functions, such as surveying the contents of the lumen and protecting from infection, increasing gut motility, modifying mucin production and other defending secretions, as well as stimulating IgA production [27].

Moreover, in the present study higher villi height in duodenum, jejunum and ileum were observed when chicks were fed diets supplemented with 0.1 (I3) and 0.125 g/kg (I4) of inulin. To this regard, it is well known that villus height play a key role in the efficacy of digestion and absorption processes; in fact, higher villi increase the absorption surface area, with that enhancing the absorption of dietary nutrients [28]. Hence, together to the improved growth performances, this seems to explain the general positive effect observed because of the inulin diet inclusion. Several researchers have reported similar results; first of all, Rehman et al [14] already observed that longer jejunum villi in chicks were obtained when diets were supplemented with 0.1 g/kg of inulin; similarly, Awad et al [11] described changes in the mucosal architecture in terms of increased ileal villi height in birds fed a diet supplemented with 0.01 g/kg symbiotic mixture containing *Enterococcus faecium* and oligosaccharides; Sayrafi et al [23] observed that a 0.01 g/kg prebiotic (derived from *Saccharomyces cerevisiae* cell walls, mainly consisting in mannan oligosaccharide and β-glucan) significantly increased the villi length and width at duodenum and the ileum level.

More contrasting results are referred for crypt depth; in fact, while Awad et al [11] and Sayrafi et al [23] observed shorter depth of the crypts in the duodenum and ileum of chickens fed prebiotics (inulin and *Saccharomyces cerevisiae* cell wall, respectively), Rehman et al [14] found that 0.1 g/kg inulin-supplemented diet resulted in deeper crypts of villi in chickens. In the present study, results are in agreement with the observations described by Rehman et al [14] since increases of crypt depth were observed at duodenum, jejunum and ileum levels when birds were fed diets containing 0.1 and 0.125 g/kg inulin.

The current study demonstrated that the number of goblet cells of duodenum, jejunum and ileum was increased by feeding birds a diet supplemented with 0.075, 0.1, 0.125, and 0.15 g/kg inulin. To this regard, Pelicano et al [13] agree that the addition of prebiotics can increase the number of goblet cells in the intestines of chickens. The improvement in crypt depth and in the density of goblet cells observed in birds treated with prebiotic may be due to a rapid crypt maturation to assure an adequate epithelium turnover rate [13].

While an increase in the number and type of beneficial species of microbes has recently been used in the probiotic treatment in animals to improve nutrition and health, macromolecules synthesized by some microorganisms are also being employed as prebiotic treatments [19]. Prebiotics are defined as food ingredients that selectively stimulate the growth and activity of beneficial microorganisms such as *Bifidobacterium* and *Lactobacillus* in the gut and thereby can benefit animal health [4,27,28]. According to this vision, in the present study it was observed that the administration of 0.1 g/kg dietary inulin to chicks significantly decreased the concentration of *Escherichia coli* in cecum, while increasing the concentration of *Bifidobacterium* and *Lactobacillus* in cecum and ileum. In a similar study, Tako et al [29] found a significant variation in relative amounts of *Bifidobacterium* and *Lactobacillus* in the intestinal content within their treatments’ groups, and relative amounts of *Bifidobacterium* was increased with increasing the prebiotic content in diet. Sharma and Kanwar [30] investigating the enhancing effects of prebiotics on intestinal microflora showed that these compounds prevent pathogenic bacteria from affecting the intestinal cell turnover rate and, at the same time, helping to curtail the establishment of pathogenic bacteria within the intestinal cells.

Finally, in relation to the effects of prebiotics and more specifically inulin, on ascites incidence in chicks, the results of the present study are in agreement with those reported by Solis de los Santos et al [17]; in fact, these authors found that broilers fed with prebiotic and reared under hypoxic conditions, showed a 23% lower incidence of ascites compared with untreated birds raised in the same environment.

In conclusion, the results of the present study clearly suggest that several parameters such as body weight and body weight gain, feed intake, intestinal morphology and microbial balance, may be significantly enhanced by supplementing a diet for broilers with inulin; moreover, the incidence of ascites can also be drastically reduced even when chicks are raised at a high altitude and hypoxic conditions. Hence, according to the results of the study it is suggested to include inulin in chicks’ diets at a rate ranging between 0.1 and 0.125 g/kg.

These findings may be of particular interest for the poultry industry globally, but they represent a relevant opportunity for those regions characterized by high altitude. This is the case of some Western China regions where these results may be of relevance to its poultry meat production.

## Figures and Tables

**Table 1:** The dietary composition of feed ingredients supplied to Haidong chicks

Items	C1)	Inulin1) (0 to 3 wk)					C1)	Inulin1) (4 to 6 wk)				
	
		I1	I2	I3	I4	I5		I1	I2	I3	I4	I5
Ingredients (%)												
Maize	52.77	52.77	52.77	52.77	52.77	52.77	48.77	48.77	48.77	48.77	48.77	48.77
Soybean meal	36.0	36.0	36.0	36.0	36.0	36.0	40.7	40.7	40.7	40.7	40.7	40.7
Wheat bran	3.0	2.5	2.25	2.0	1.75	1.5	3	2.5	2.25	2.0	1.75	1.5
Soybean oil	3.6	3.6	3.6	3.6	3.6	3.6	3	3	3	3	3	3
Limestone	1.16	1.16	1.16	1.16	1.16	1.16	1.06	1.06	1.06	1.06	1.06	1.06
Inulin	0	0.5	0.75	1.0	1.25	1.5	0	0.5	0.75	1.0	1.25	1.5
Met	0.2	0.2	0.2	0.2	0.2	0.2	0.2	0.2	0.2	0.2	0.2	0.2
Lys	0.02	0.02	0.02	0.02	0.02	0.02	0.02	0.02	0.02	0.02	0.02	0.02
Vitamin-mineral mix2)	1.0	1.0	1.0	1.0	1.0	1.0	1	1	1	1	1	1
Sodium chloride	0.3	0.3	0.3	0.3	0.3	0.3	0.3	0.3	0.3	0.3	0.3	0.3
Dicalcium phosphate	1.95	1.95	1.95	1.95	1.95	1.95	1.95	1.95	1.95	1.95	1.95	1.95
Nutrient composition calculated												
ME (kcal/kg)	2,988	2,986	2,984	2,982	2,980	2,981	2,914	2,892	2,881	2,870	2,859	2,848
Nutrient composition analyzed (%)												
Dry matter (%)	89.5	89.8	89.4	89.6	89.3	89.6	90.2	90.2	90.3	90.3	90.3	90.3
CP (%)	20.8	20.6	20.6	20.5	20.4	20.3	22.3	22.1	22.0	21.9	21.9	21.8
Fat (%)	2.4	2.4	2.4	2.4	2.4	2.4	2.4	2.3	2.3	2.3	2.3	2.3
Met (%)	0.64	0.64	0.64	0.64	0.64	0.64	0.66	0.66	0.66	0.66	0.66	0.66
Lys (%)	1.24	1.23	1.24	1.23	1.23	1.23	1.37	1.36	1.36	1.36	1.35	1.35
Ca (%)	0.98	0.98	0.98	0.98	0.98	0.98	0.97	0.97	0.97	0.97	0.96	0.96
Av. P (%)	0.49	0.49	0.49	0.49	0.49	0.49	0.51	0.50	0.49	0.49	0.48	0.48

ME, metabolizable energy; CP, crude protein.

1) C: control group, no inulin was added; for a contrary; I1, I2, I3, I4, I5: to the diet fed to the remaining groups, 0.05, 0.075, 0.1, 0.125 and 0.15 g/kg inulin.

2) Supplied per kilogram of diet: vitamin A, 15,000 IU; vitamin D_3_, 3,000 IU; vitamin E, 26 IU; vitamin K, 2.5 mg; vitamin B_1_, 2.1 mg; vitamin B_2_, 5 mg; vitamin B_6_, 3 mg; vitamin B_12_, 6 mg; folic acid, 1 mg; biotin, 50 μg; niacin, 24 mg; cholinechloraid, 50 mg, manganeze, 45 mg, iron, 83mg; zinc, 10 mg, copper, 0.26 mg; iodine, 1.5 mg; selenium, 1.2 mg.

**Table 2:** The effect of dietary inulin on growth performances, feed intake, and feed conversion ratio

Group1)	BW (g)		WG (g)			FI (g)			FCR
			
	1 d	42 d	1 to 21 d	22 to 42 d	1 to 42 d	1 to 21 d	22 to 42 d	1 to 42 d	1 to 42 d
C	39.52	1,942.32^c^	626.60	1,276.20^c^	1,902.80^c^	1,089.32^c^	3,122.43^d^	4,211.75^e^	2.21
I1	39.48	1,948.67^c^	635.99	1,273.20^c^	1,909.19^c^	1,106.48^b^	3,136.64^cd^	4,243.12^cd^	2.22
I2	39.33	1,972.54^ab^	640.86	1,292.35^ab^	1,933.21^ab^	1,113.36^ab^	3,159.51^ab^	4,272.87^b^	2.21
I3	39.26	1,983.58^a^	656.12	1,288.20^ab^	1,944.32^a^	1,123.22^a^	3,178.38^a^	4,301.60^a^	2.21
I4	39.78	1,976.79^a^	638.63	1,298.38^a^	1,937.01^a^	1,109.37^b^	3,161.27^ab^	4,270.64^b^	2.20
I5	39.63	1,956.35^bc^	634.95	1,281.77^bc^	1,916.72^bc^	1,114.84^ab^	3,148.21^bc^	4,263.05^bc^	2.22
SEM	1.39	73.48	22.49	51.96	74.45	42.35	123.97	135.56	0.088
p-value	0.853	0.016	0.003	0.008	0.011	0.041	0.029	0.024	0.695

BW, body weight; WG, weight gain; FI, feed intake; FCR, feed conversion ratio; SEM, standard error of the mean.

1) C: control group, no inulin was added; for a contrary; I1, I2, I3, I4, I5: to the diet fed to the remaining groups, 0.05, 0.075, 0.1, 0.125 and 0.15 g/kg inulin.

a–e Means within columns with different superscript letters are different per p<0.05.

**Table 3:** The effect of dietary inulin level on blood parameters

Treatments1)	PCV (%)		RBC (10^6^×cell/mm^3^)		WBC (10^3^×cell/mm^3^)		Hb (%)	
			
	21 d	42 d	21 d	42 d	21 d	42 d	21 d	42 d
C	40.5^c^	47.6^c^	2.98^d^	3.21^d^	21.32^c^	23.41	114.2^d^	104.5^d^
I1	47.6^ab^	55.8^a^	3.49^bc^	3.92^a^	23.43^bc^	23.63	127.8^c^	118.5^c^
I2	47.9^ab^	52.5^ab^	3.43^c^	3.86^ab^	22.61^bc^	24.32	132.6^b^	124.2^ab^
I3	46.4^ab^	52.9^ab^	3.88^a^	3.62^bc^	24.45^ab^	25.98	127.5^c^	121.6^bc^
I4	49.8^a^	55.7^a^	3.53^bc^	3.86^ab^	25.79^a^	25.48	138.3^a^	126.2^a^
I5	43.3^bc^	50.8^bc^	3.90^a^	3.95^a^	21.14^c^	23.64	136.9^ab^	125.5^a^
SEM	1.46	1.87	0.13	0.12	0.89	0.95	4.63	4.34
p-value	0.014	0.026	0.034	0.041	0.009	0.893	0.006	0.018

PCV, packed cell volume; RBC, red blood cell; WBC, white blood cell; Hb, hemoglobin; SEM, standard error of the mean.

1) C: control group, no inulin was added; for a contrary; I1, I2, I3, I4, I5: to the diet fed to the remaining groups, 0.05, 0.075, 0.1, 0.125 and 0.15 g/kg inulin.

a–d Means within columns with different superscript letters are different per p<0.05.

**Table 4:** Effects of dietary inulin on intestinal wall and muscle thickness

Group1)	Intestinal wall thickness (μm)			Intestinal muscle thickness (μm)		
	
	Duodenum	Jejunum	Ileum	Duodenum	Jejunum	Ileum
C	306.5^c^	332.6^b^	333.8^cd^	186.5^b^	130.4^c^	140.2^b^
I1	309.5^c^	347.4^b^	323.9^d^	152.7^c^	131.3^c^	150.3^ab^
I2	340.6^b^	342.1^b^	349.6^bc^	189.4^b^	132.6^c^	162.8^a^
I3	378.4^a^	391.1^a^	381.0^a^	232.7^a^	198.4^a^	152.2^ab^
I4	344.3^b^	389.4^a^	365.23^ab^	230.1^a^	165.9^b^	149.8^ab^
I5	357.6^b^	390.8^a^	372.3^a^	228.9^a^	162.9^b^	151.1^ab^
SEM	13.87	13.35	12.14	6.28	4.60	4.89
p-value	0.012	0.023	0.008	0.035	0.015	0.043

SEM, standard error of the mean.

1) C: control group, no inulin was added; for a contrary; I1, I2, I3, I4, I5: to the diet fed to the remaining groups, 0.05, 0.075, 0.1, 0.125 and 0.15 g/kg inulin.

a–d Means within columns with different superscript capital letters at same day are different (p<0.05).

**Table 6:** Goblet cells density (n/mm^2^) and ascites incidence (%)

Group1)	Goblet cell (cells/villus)			Ascites incidence

	Duodenum	Jejunum	Ileum	
C	36.23^c^	39.55^c^	65.11^d^	32.34^a^
I1	50.65^bc^	56.82^b^	78.24^c^	25.46^b^
I2	63.17^ab^	62.98^b^	95.51^b^	27.64^b^
I3	70.42^a^	77.65^a^	112.55^a^	27.71^b^
I4	69.49^a^	79.94^a^	106.88^ab^	27.82^b^
I5	68.66^a^	78.89^a^	105.63^ab^	26.73^b^
SEM	2.16	2.19	3.79	1.12
p-value	0.015	0.009	0.037	0.040

SEM, standard error of the mean.

1) C: control group, no inulin was added; for a contrary; I1, I2, I3, I4, I5: to the diet fed to the remaining groups, 0.05, 0.075, 0.1, 0.125 and 0.15 g/kg inulin.

a–d Means within columns with different superscript capital letters at same day are different (p<0.05).

**Table 5:** Effect of dietary inulin level on intestinal villus height and crypt depth

Group1)	Height of intestinal villus (μm)			Depth of crypt (μm)			Villus height/ villus crypt values		
		
	Duodenum	Jejunum	Ileum	Duodenum	Jejunum	Ileum	Duodenum	Jejunum	Ileum
C	1,443.6^c^	1,213.9^c^	616.1^c^	165.3^b^	146.2^b^	112.3^b^	8.73	8.30	5.49
I1	1,454.3^c^	1,352.6^b^	605.2^c^	168.4^b^	156.6^ab^	116.5^b^	8.64	8.64	5.19
I2	1,523.6^b^	1,343.7^b^	687.1^b^	202.8^a^	163.1^ab^	137.2^a^	7.51	8.24	5.01
I3	1,578.2^a^	1,387.1^a^	752.5^a^	212.3^a^	170.4^a^	139.6^a^	7.43	8.14	5.39
I4	1,535.7^b^	1,378.3^a^	742.4^a^	206.8^a^	169.4^a^	143.1^a^	7.43	8.14	5.19
I5	1,531.3^b^	1,383.4^a^	738.9^a^	205.5^a^	168.6^a^	140.2^a^	7.45	8.21	5.27
SEM	44.21	41.73	18.69	6.36	5.40	4.95	0.095	0.073	0.078
p-value	0.002	0.016	0.004	0.029	0.046	0.022	0.001	0.002	0.001

SEM, standard error of the mean.

1) C: control group, no inulin was added; for a contrary; I1, I2, I3, I4, I5: to the diet fed to the remaining groups, 0.05, 0.075, 0.1, 0.125 and 0.15 g/kg inulin.

a–c Means within columns with different superscript capital letters at same day are different (p<0.05).

**Table 7:** The bacterial species composition in cecum and ileum using various supplemental feed regimes in broilers

Group1)	Cecum			Ileum		
	
	*Escherichia coli*	Bifidobacterium	Lactobacillus	*Escherichia coli*	Bifidobacterium	Lactobacillus
C	8.32^a^	7.33^b^	8.02^b^	6.48	6.03^b^	5.63^b^
I1	8.23^a^	7.78^ab^	9.67^a^	6.42	6.41^ab^	6.72^a^
I2	7.12^b^	8.36^a^	9.33^a^	6.32	6.59^ab^	6.52^a^
I3	7.01^b^	8.39^a^	9.48^a^	6.15	6.76^ab^	6.87^a^
I4	7.59^ab^	8.46^a^	9.15^a^	6.11	7.21^a^	7.01^a^
I5	7.26^b^	8.29^ab^	9.24^a^	6.21	7.25^a^	6.89^a^
SEM	0.19	0.23	0.20	0.30	0.34	0.36
p-value	0.006	0.035	0.012	0.695	0.042	0.037

SEM, standard error of the mean.

1) C: control group, no inulin was added; for a contrary; I1, I2, I3, I4, I5: to the diet fed to the remaining groups, 0.05, 0.075, 0.1, 0.125 and 0.15 g/kg inulin.

a,b Means within columns with different superscript capital letters at same day are different (p<0.05).
